# Supplementary figures and images for: Vps60 initiates alternative ESCRT-III filaments
Source: J Cell Biol. 2023 Sep 28;222(11):e202206028. doi: 10.1083/jcb.202206028 (PMC10538557; doi:10.1083/jcb.202206028)

Vps60-HA

*VPS4*

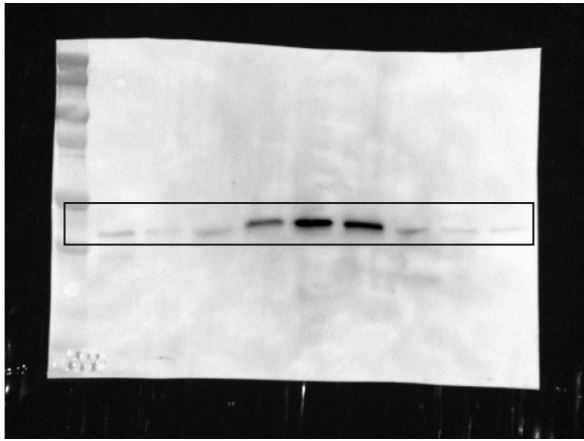

Snf7

*VPS4*

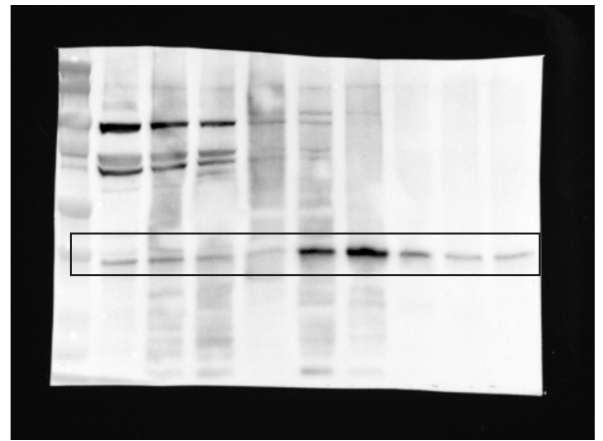

Vps60-HA

*vps4Δ*

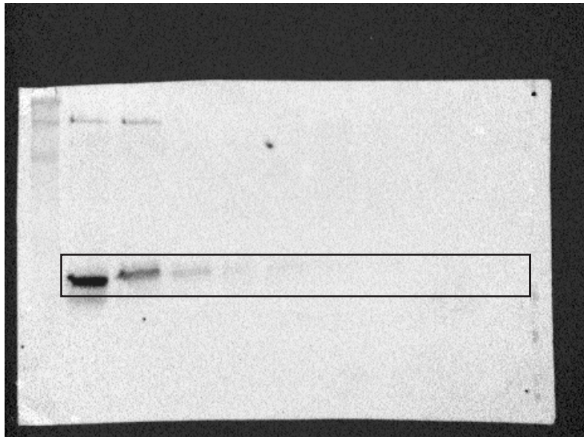

Snf7

*vps4Δ*

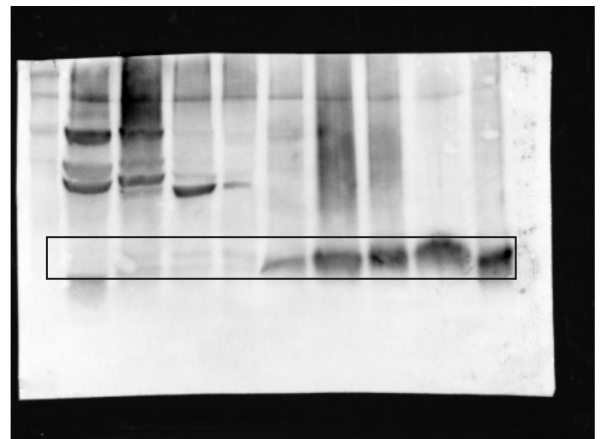

Vps60-HA

*vps4<sup>E233Q</sup>*

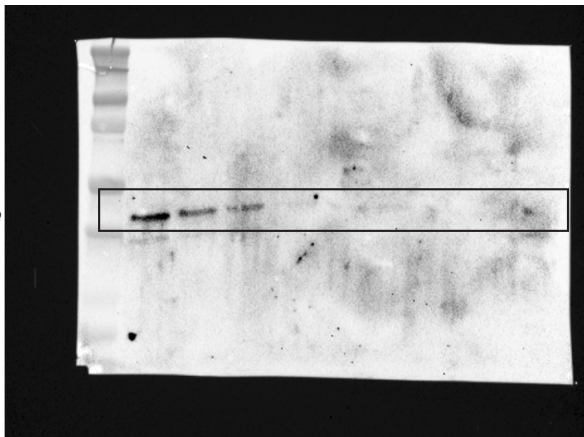

Snf7

*vps4<sup>E233Q</sup>*

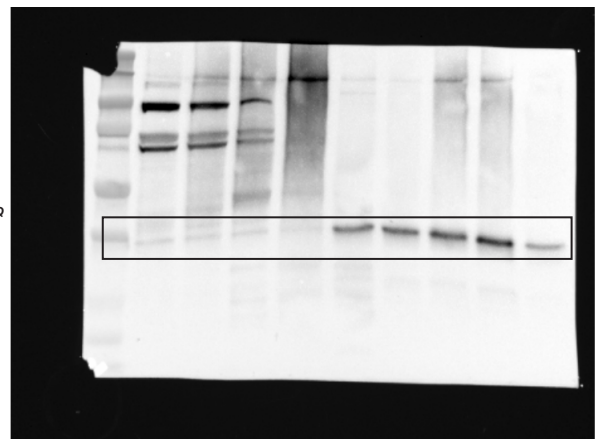

Supplement: SourceData F6 — is the source file for Fig. 6. [file JCB_202206028_SourceDataF6.pdf]

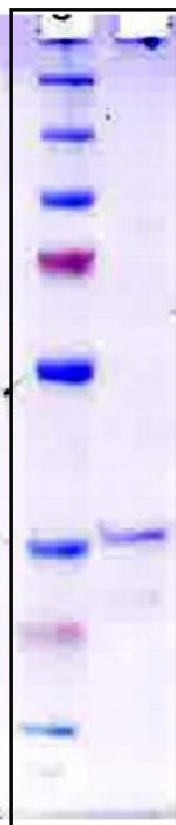

Supplement: SourceData FS1 — is the source file for Fig. S1. [file JCB_202206028_SourceDataFS1.pdf]
